# Human T-Lymphoid Progenitors Generated in a Feeder-Cell-Free Delta-Like-4 Culture System Promote T-Cell Reconstitution in NOD/SCID/γc^−/−^ Mice

**DOI:** 10.1002/stem.1145

**Published:** 2012-07-24

**Authors:** Christian Reimann, Emmanuelle Six, Liliane Dal-Cortivo, Andrea Schiavo, Kevin Appourchaux, Chantal Lagresle-Peyrou, Corinne de Chappedelaine, Brigitte Ternaux, Laure Coulombel, Kheira Beldjord, Marina Cavazzana-Calvo, Isabelle Andre-Schmutz

**Affiliations:** 1 U768 INSERM, Université Paris Descartes, Sorbonne Paris Cité, Faculté de MédecineParis, France; 2 Fondation IMAGINEParis, France; 3 Center for Pediatrics & Adolescent Medicine, University Medical CenterFreiburg, Germany; 4 Département de Biothérapie, AP-HP, Hopital Necker-Enfants MaladeParis, France; 5 CIC-BT 502 INSERMParis, France; 6 U935 INSERMVillejuif, France; 7 Laboratoire Central d'Hématologie, AP-HP, Hopital Saint LouisParis, France

**Keywords:** Hematopoietic stem cell transplantation, Immunotherapy, T-lymphoid precursor cells, DLL4 protein, Notch1

## Abstract

Slow T-cell reconstitution is a major clinical concern after transplantation of cord blood (CB)-derived hematopoietic stem cells. Adoptive transfer of in vitro-generated T-cell progenitors has emerged as a promising strategy for promoting de novo thymopoiesis and thus accelerating T-cell reconstitution. Here, we describe the development of a new culture system based on the immobilized Notch ligand Delta-like-4 (DL-4). Culture of human CD34^+^ CB cells in this new DL-4 system enabled the in vitro generation of large amounts of T-cell progenitor cells that (a) displayed the phenotypic and molecular signatures of early thymic progenitors and (b) had high T lymphopoietic potential. When transferred into NOD/SCID/γc^−/−^ (NSG) mice, DL-4 primed T-cell progenitors migrated to the thymus and developed into functional, mature, polyclonal αβ T cells that subsequently left the thymus and accelerated T-cell reconstitution. T-cell reconstitution was even faster and more robust when ex vivo-manipulated and nonmanipulated CB samples were simultaneously injected into NSG mice (i.e., a situation reminiscent of the double CB transplant setting). This work provides further evidence of the ability of in vitro-generated human T-cell progenitors to accelerate T-cell reconstitution and also introduces a feeder-cell-free culture technique with the potential for rapid, safe transfer to a clinical setting.

## INTRODUCTION

Unrelated cord blood transplantation (UCBT) has become an important therapeutic procedure for patients lacking human leukocyte antigen (HLA)-compatible donors [1]. Although originally conceived for hematopoietic stem cell transplantation (HSCT) in children, the use of double cord blood (CB) units circumvents the limitation of low cell doses and makes double UCBT (dUCBT) a valid option in adults. However, serious infections constitute the leading nonmalignant cause of post-transplant morbidity and mortality and are undoubtedly related to delayed T-cell immune reconstitution and impaired de novo thymopoiesis [2–5]. Donor-derived T cells generated in the recipients thymus first appear 9 months after UCBT [1]. Complete restoration of the T-cell compartment with a polyclonal T-cell repertoire takes even longer and may never reach pretransplant levels [3, 6].

After HSCT, de novo T-cell generation from donor hematopoietic progenitors is disturbed at several successive steps. First, the generation of T-cell progenitors in the bone marrow (BM) and their delivery to the thymus have been shown to represent a limiting step in post-HSCT recovery [7]. Furthermore, conditioning regimens, graft versus-host-disease, infectious disease, and inflammatory status damage the thymic stroma and thereby alter intrathymic T-cell differentiation [8].

A promising approach for accelerating de novo T-cell development consists in bypassing T-cell precursor delivery from the BM by adoptively transferring in vitro-generated T-lymphoid progenitors. The latter should be capable of immediately seeding the thymus and then rapidly generating a wave of donor-derived, polyclonal, host-tolerant T cells.

Notch-1 signaling plays a major role for priming human stem cells (HSCs) toward the T-cell lineage and thus for early thymopoiesis [9–11]. Notch-based culture systems, such as delta-like-1 (DL-1)-expressing BM stromal cells (OP9/DL-1 cells), allow in vitro generation of large numbers of human T-cell progenitors that have been demonstrated to promote de novo thymopoiesis in humanized mice [12–14]. This working hypothesis was further supported by observations in conventional mouse models: when transferred into allogeneic recipients, murine T-cell progenitors generated in OP9/DL-1 coculture accelerated de novo thymopoiesis and emergence of host-tolerant, functional T cells that afforded protection against infectious agents and residual tumor cells when transferred into an allogeneic HSCT model [15, 16].

With a view to establish a clinically applicable system, feeder-cell-free Notch-ligand culture systems for the generation of T-lymphopoietic progenitors are warranted. Replacement of the OP9/DL-1 cells with the Fc-fusion-protein DL-1/Fc enabled high-grade expansion of hematopoietic progenitors in vitro. These progenitors were found to enhance bone marrow engraftment and myeloid reconstitution in xenotransplantation models and in a recent phase I clinical trial [17, 18]. Unfortunately, this procedure does not appear to have an influence on the kinetics of T-cell reconstitution.

Recent studies have shown that (a) DL-4 is the essential Notch 1 activator in the T-engagement of hematopoietic stem cells [19–21] and (b) immobilized DL-4 induces phenotypic changes reminiscent of early T-cell development in CD34^+^ CB cells having been expanded in an MS-5 culture system [22]. By extending this approach, we found that immobilized DL-4 supported the in vitro generation of T-cell progenitor cells from CD34^+^ CB cells in a feeder-cell-free culture system. We further demonstrated that DL-4 generated T-cell progenitors displayed the phenotypic and molecular signatures of very immature thymic precursors. Upon transfer in NOD/SCID/γc^−/−^ (NSG) mice, these progenitors were capable of migrating to the thymus and differentiating into mature polyclonal T cells. This work provides a basis for rapidly translating our ex vivo culture system into clinical procedures for accelerating immune reconstitution after partially HLA-mismatched, allogeneic HSCT.

## MATERIALS AND METHODS

### Human Cells and Tissues

CB samples not eligible for banking were used, following the provision of informed consent by the child's mother.

Human thymus tissue samples were obtained from children undergoing heart surgery. The samples were collected as previously described [23] and used in accordance with the French legislation and ethical guidelines.

### Cell Preparation

CD34^+^ hematopoietic progenitors were magnetically sorted from human CB and thymus, as previously described [23]. For quantitative polymerase chain reaction (PCR) experiments, CD34^+^ cells were further sorted on an ARIA II system (BD Biosciences, San José, CA) to exclude residual CD3^+^, CD56^+^, and CD19^+^ cells.

### Generation of the Delta-4 Fc Fusion Protein and a Control IgG2b Fc-Fragment

The Delta-4 Fc fusion protein construct was generated by cloning cDNA encoding the soluble domain of hDL-4 (aa1-526) (kindly provided by Adrian Harris) into the pFuse-hFC1 vector (InvivoGen, San Diego, CA, http://www.invivogen.com/) containing the coding region for the human IgG2bFc fragment. The DL-4-Fc fusion construct (or the pFuse-hFc1 fragment alone) was then subcloned into an MSCV-IRES-PuroR retroviral vector and transduced into HEK293T cells. The DL-4-Fc fusion protein and the control IgG2b Fc-fragment were produced and purified by PX'Therapeutics (Grenoble, France, http://www.px-therapeutics.com/).

### Exposure of CD34^+^ Progenitor Cells to Notch Ligand DL-4

CD34^+^ CB cells were cultured in 24-well plates previously coated with DL-4 (5 μg/ml) or control-Fc (5 μg/ml) for 24 hours at 4°C, blocked with bovine serum albumin 2% in phosphate-buffered saline (PBS) for 1 hour at 37°C, and washed with PBS. Cultures were initiated at a concentration of 2 × 10^4^ cells per well in X-VIVO 20 medium (BioWhittaker, Walkersville, MD), supplemented with 20% defined fetal calf serum (Hyclone, Thermo Fisher Scientific, Illkirch, France) and the recombinant human cytokines interleukin-7 (IL-7), Flt3-ligand (Flt-3), stem cell factor, and thrombopoietin (all at 100 ng/ml and all purchased from either R&D Systems [Minneapolis, MN, http://www.rndsystems.com] or PeproTech Inc. [Rocky Hill, NJ, http://www.peprotech.com]). If the culture lasted longer than 1 week, cells were transferred into freshly coated wells at a maximum concentration of 1 × 10^5^ cells per well. After 7 days of culture on DL-4, cultured cells were sorted by fluorescence-activated cell sorting (FACS) to exclude CD34^−^/CD7^−^ myeloid cells from subsequent analyses. The cells in the resulting fraction are referred to hereafter as “DL-4 cells.”

### The OP9/DL-1 In Vitro T-Cell Differentiation Assay

The T-lymphoid potential of native CD34^+^ CB cells and T-cell progenitors generated by exposure to DL-4 was assessed via limiting dilution assays (LDAs) in OP9/DL-1 cocultures, as previously described [24].

### Quantitative, Real-Time PCRs

Total RNA was isolated with the RNeasy Micro Kit (Qiagen, Courtaboeuf, France, http://www.qiagen.com) and then reverse-transcribed using Multiscribe reverse transcriptase and oligo(dT) primers with the high-capacity cDNA Reverse Transcription Kit (Life Technologies, Carlsbad, CA, http://www.lifetechnologies.com). Diluted cDNA was used as a template for quantitative, real-time PCR reactions in TaqMan gene expression assays for Pre-T-cell Receptor alpha (*PT*α), interleukin-7 receptor alpha (*IL7R*α), recombination activating gene 1 (*RAG1*), B-cell lymphoma/leukemia 11B (*BCL11B*), spleen focus forming virus proviral integration oncogene 1 (*SPI-1*), and paired box protein Pax-5 (*PAX5*) (Applied Biosystems, US, http://appliedbiosystems.com). The PCRs were performed on an ABI Prism 7900 system (Applied Biosystems, US, http://appliedbiosystems.com), according to the manufacturer's instructions. All transcript levels were normalized against the results for human *glyceraldehyde-3-phosphate dehydrogenase*.

### T-Cell Receptor Recombination Analysis

T-cell receptor (TCR) recombination analysis of in vitro-generated DL-4 cells and native human thymocytes was performed as previously described [25]. The TCR-β-rearrangement repertory of human T cells recovered from the thymuses of transplanted NOD/SCID/γc^−^/^−^ mice (referred to here as “NSG” mice) was measured using the ImmunTraCkeR assay (ImmunID Technologies, Grenoble France, http://www.immunid.com/), as previously described [26].

### Flow Cytometry Analysis and Cell Sorting

Monoclonal antibodies against CD1a (HI149), CD3 (SK7), CD4 (SK3), CD5 (UCHT2), CD7 (M-T701), CD8 (RPA-T8), CD16 (Leu11c), CD34 (8G12), CD45, CD45RA (HI100), CD56 (My31), CXCR4 (12G5), and 7-aminoactinomycin D (7AAD) were obtained from BD Biosciences. Monoclonal antibodies against TCRαβ (IP26A) and TCRγδ (IMMU510) were obtained from Beckman Coulter (Brea, CA, https://www.beckmancoulter.com). Cell suspensions were stained and analyzed on an 8-color FACSCanto II cytometer (BD Biosciences, http://www.bdbiosciences.com). The data were analyzed using FlowJo software (Treestar, Ashland, OR, http://www.flowjo.com/) after gating on viable, 7AAD-negative cells. Absolute cell numbers were determined using Caltag counting beads (Invitrogen, Camarillo, CA, http://www.invitrogen.com). Cell subsets were sorted on an ARIA II system.

### Adoptive Transfer of In Vitro-Generated T-Cell Progenitors into NSG Mice

The NSG mice (obtained from The Jackson Laboratory, Bar Harbor, ME, http://www.jax.org) were housed in a pathogen-free facility. DL-4 cells (5 × 10^5^) were injected intrahepatically into newborn, nonirradiated NSG mice or intravenously into adult (4-week old), 3Gy-irradiated NSG mice. Control mice were injected with either 1.5 × 10^5^ noncultured CD34^+^ cells or 1 × 10^6^ cells cultured on immobilized control-Fc molecules. Regardless of the type of cells injected (i.e., DL-4 cells, control-Fc cells, or noncultured CD34^+^ cells), each animal received a total of 1.5 × 10^5^ CD34^+^/CD7^−^ cells.

Transplanted mice were subcutaneously injected with 5 mg of recombinant human IL-7 (kindly provided by Cytheris, Issy-les-Moulineaux, France, http://www.cytheris.com/) every 7 days. Nonirradiated newborn NSG mice were sacrificed at 1, 2, 3, or 4 weeks and 3 Gy-irradiated adult NSG mice at 8 weeks post-transplant. Cells were harvested from the femur, spleen, thymus, and peripheral blood and analyzed by flow cytometry, as described above. All experiments and procedures were performed in compliance with the French Ministry of Agriculture's regulations on animal experiments.

### Cytokine Production

Peripheral blood mononuclear cells from spleens were obtained after mechanic disruption and red blood cell lysis. Splenocytes were cultured in the presence of 100 U/ml IL-2 and 5 ng/ml IL-7 for 7 days. The cells were then stimulated with phorbol myristate acetate (PMA) (50 ng/ml), ionomycin (1 mg/ml), and Golgi-Stop reagent for 3 hours. Intracellular IFN-γ was measured using standard flow cytometry procedures.

### Statistical Analysis

Nonparametric Mann-Whitney *U* tests were performed using Prism 4 software (GraphPad Software Inc., LA Jolla, CA, http://www.graphpad.com).

## RESULTS

### In *Vitro* Exposure of CB CD34^+^ Cells to a DL-4 Fusion Protein Induces Phenotypic Changes that are Consistent with Early T-Cell Development

**Figure 1:**
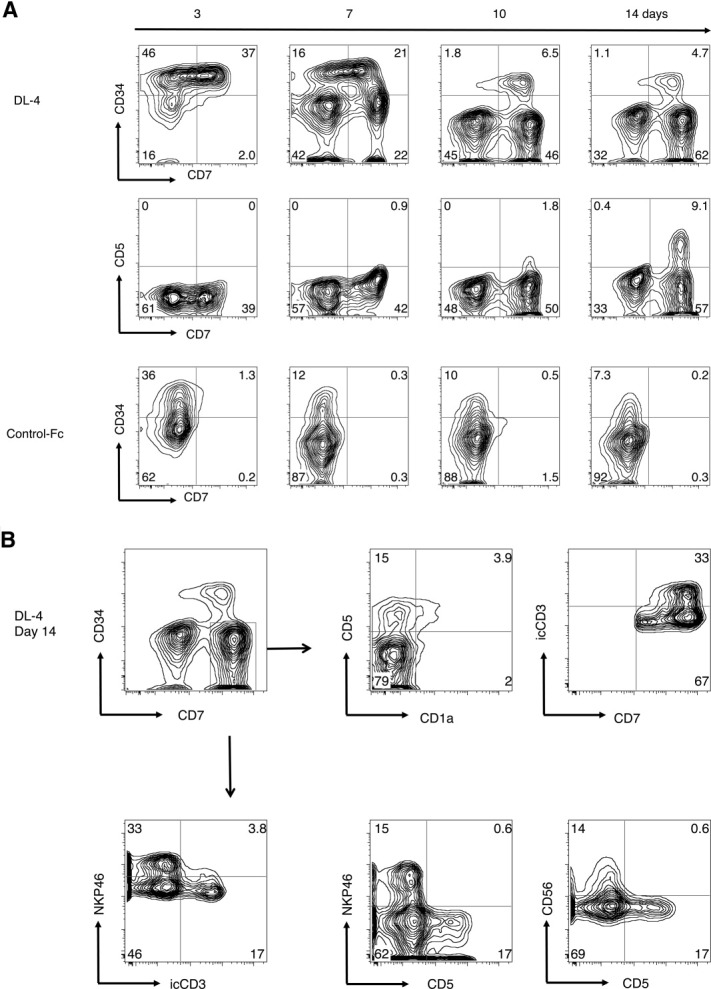
Emergence of CD7^+^ cells after exposure of CD34^+^ cells to immobilized delta4. (A): CD34^+^ cord blood cells were plated into dishes precoated with either DL-4 (upper lines) or control-Fc (lower line) and cultured for 14 days. Cultured cells were analyzed by flow cytometry for surface expression of CD34, CD7, and CD5 (as early markers of T-cell commitment) at the indicated time points. (B): Flow cytometry analysis of DL-4 cells from a 14-day culture. CD34^−^/CD7^++^ cells were analyzed for surface expression of CD5, CD1a, NKP46, CD56, and intracellular expression of CD3epsilon. Abbreviation: DL-4, delta-like-4.

Purified CD34^+^ CB cells cultured with DL-4-Fc fusion protein (DL-4) began to express CD7 within 3 days (Fig.1A, upper panel). This expression paralleled the upregulation of CD45RA (Supporting Information Fig. S1, middle panel). CD7 expression continued to increase until day 7 and was correlated with a decrease in CD34 expression and the emergence of a CD34^−^/CD7^++^ population. A T-cell progenitor subset expressing CD5 (Fig.1A medium panel), intracellular CD3epsilon (Fig.1B upper panel), and CXCR4 (Supporting Information Fig. S1 lower panel) emerged from within the CD34^−^/CD7^++^ population between days 7 and 10. By day 14, the CD34^−^/CD7^++^/CD5^+^ population had started to express low levels of CD1a (Fig.1B, upper panel). In human postnatal thymocytes, the early thymic progenitor (ETP) (CD34^+^/CD45RA^+^/CD7^+^), proT1 (CD7^++^/CD5^−^), proT2 (CD7^++^/CD5^+^), and preT stages (CD7^++^/CD5^+^CD1a^+^) mark successive T-cell developmental stages before beta selection [12]. Since we observed the characteristic expression of these antigens in DL-4 culture, our CD34^+^/CD45RA^+^/CD7^+^, CD7^++^/CD5^−^, CD7^++^/CD5^+^, and CD7^++^/CD5^+^/CD1a^+^ subsets will be referred to hereafter as ETP, proT1, proT2, and preT cells.

The lack of any CD4, CD8, surface CD3, or TCR expression in DL-4 cultures indicated that a T-cell development was blocked at this stage. A subset of the CD34^−^/CD7^++^-population was found to coexpress NKP46 and CD56 at day14, indicating differentiation toward a natural killer (NK) lineage. Phenotypically, the NK- and the T-lineage-engaged populations could be clearly distinguished from each other by mutually exclusive expression of NK-precursor markers (i.e., NKP46 and CD56) and T-precursor markers (i.e., CD5 and intracellular CD3) (Fig.1B, lower panel). In line with this differential marker expression, the NK-precursor population did not express CXCR4 (Fig. S1B, lower line).

CD34^−^/CD7^−^ cells had a myeloid phenotype and were excluded by FACS from all subsequent analyses. The remaining DL-4 fraction thus contained CD34^+^/CD7^−^, ETP, and proT1 cells. In contrast, CD34^+^ CB cells exposed to the control IgG2b Fc-fragment (“control-Fc cells”) never gave rise to CD7^+^ T-cell progenitors (Fig.1A, lower panel). The vast majority of control-Fc cells had a myeloid phenotype (data not shown) and only a small proportion was CD34^+^.

In quantitative terms, 2 × 10^4^ CD34^+^ cells (containing only 170 ETP cells) gave rise to an average of 5.0 × 10^4^ ETP-cells after 7 days of culture (Table 1, third row). This count did not change thereafter, whereas the mean number of proT1 and proT2 cells increased from 5.6 × 10^4^ on day 7 to 4.1 × 10^5^ after 14 days of DL-4 culture (data not shown).

**Table 1:** Exposure to DL-4 increases the T-cell precursor frequency of CD34^+^ cells

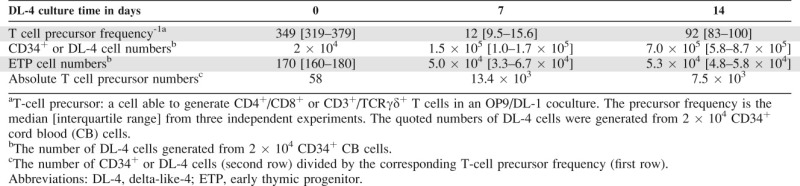

a T-cell precursor: a cell able to generate CD4^+^/CD8^+^ or CD3^+^/TCRγδ^+^ T cells in an OP9/DL-1 coculture. The precursor frequency is the median [interquartile range] from three independent experiments. The quoted numbers of DL-4 cells were generated from 2 × 10^4^ CD34^+^ cord blood (CB) cells.

b The number of DL-4 cells generated from 2 × 10^4^ CD34^+^ CB cells.

c The number of CD34^+^ or DL-4 cells (second row) divided by the corresponding T-cell precursor frequency (first row).

Abbreviations: DL-4, delta-like-4; ETP, early thymic progenitor.

### DL-4 Cells Display the Molecular Characteristics of Early T-Cell Progenitors

**Figure 2:**
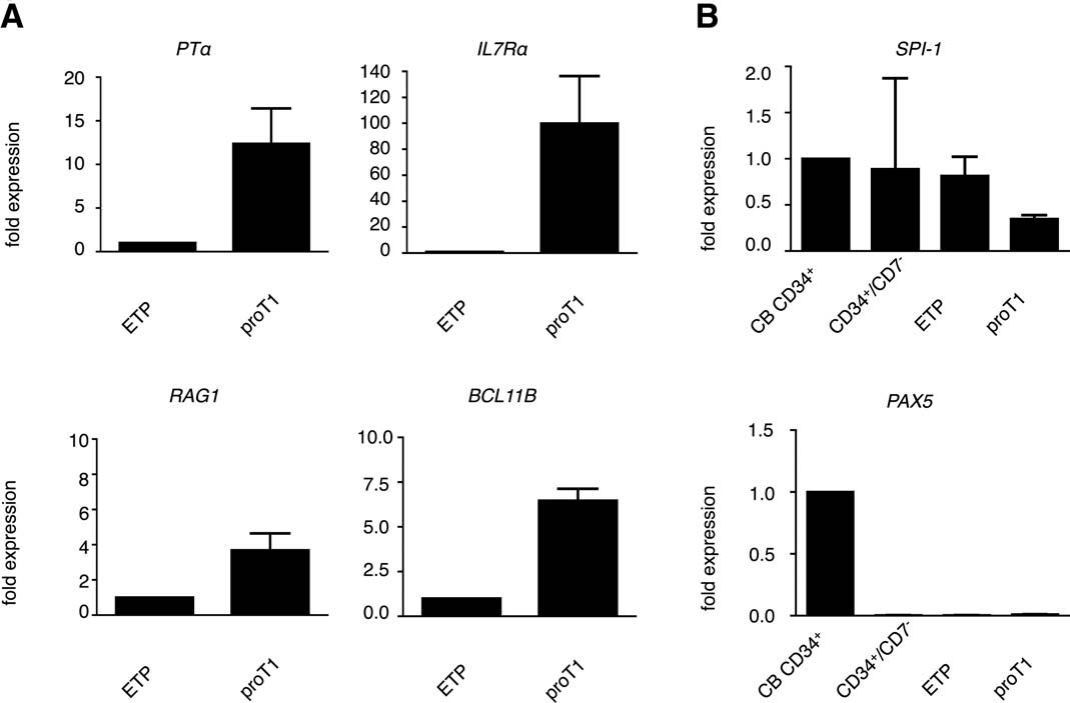
The gene expression profile of delta-like-4 (DL-4) cell subsets. (A): TaqMan polymerase chain reaction gene expression analysis in CD34^+^/CD7^−^, ETP, and proT1 DL-4 subsets generated after 7 days of culture. Transcript levels for the indicated genes were normalized against human GAPDH expression. Expression of T-cell developmental genes within the CD34^+^/CD7^−^ subset was either not detected (*PT*α) or negligible (*IL7Rα, RAG1*, and *BCL11B*). Levels found in the proT1 population were compared with those in the ETP subset. The columns represent the median-fold expression values obtained in three independent experiments. Bars indicate the interquartile range. (B): Transcript levels for the indicated genes were normalized against human *GAPDH* expression. Gene expression levels found in DL-4-primed cell populations were compared with the initial expression levels of *SPI-1* and *PAX5* in noncultured CD34^+^ cells. The columns represent median-fold expression values obtained in three independent experiments. Bars indicate the interquartile range. Abbreviations: BCL11B, B-cell lymphoma/leukemia 11B; ETP, early thymic progenitor; IL7Rα, interleukin 7 receptor alpha; PTα, pre-T-cell receptor alpha; RAG1, recombination activating gene 1; SPI-1, spleen focus forming virus proviral integration oncogene 1, PAX5, paired box protein 5; GAPDH, glyceraldehyde-3-phosphatase dehydrogenase.

To confirm that acquisition of the ETP and proT1 phenotype had opened (and were actively transcribing) a T-cell differentiation program after 7 days of DL-4 culture, we studied the cells' expression of genes involved in early T-cell development and compared them with the CD34^+^/CD7^-^ subset. The latter did not express the *PT*α, *IL7R*α, *RAG1*, and *BCL11B* genes (data not shown). In contrast, all these genes were initially expressed in ETP cells and substantially upregulated in proT1 cells. Accordingly, *SPI-1* was downregulated in the proT1 subset and *PAX5* was silenced in all subsets after DL-4 exposure (Fig.2B). Although we did not detect any TCR rearrangements after 7 days of DL-4 culture (Supporting Information Table S1), longer culture times did induce these events in a similar pattern as seen in human thymopoiesis (Supporting Information Table S1).

### DL-4-Primed ETP and proT1 Cells Have High T-Lymphoid Potential

As demonstrated above, the DL-4 cells' phenotypic and molecular characteristics were consistent with early T-cell engagement. In order to quantify the cells' in vitro T-lymphoid potential, serial dilutions obtained after 0, 7, and 14 days of cultures were placed in secondary OP9/DL-1 cocultures and analyzed in an LDA (Table 1). A cell able to complete T-cell differentiation in vitro is referred to hereafter as a “T-cell precursor.” Noncultured CD34^+^ cells displayed a T-cell precursor frequency of 1 in 349 (Table 1). Seven days of exposure to DL-4 induced a 29-fold increase in the T-cell precursor frequency (i.e., to 1 in 12). Although high numbers of proT1 and proT2 cells were generated after, a considerably lower T-cell precursor frequency was found after 14 days of culture (1 in 92); this correlated with the high number of NK-like cells observed after prolonged culture. Likewise, exposure to DL-4 produced a multilog-fold increase of T-cell precursors, whereas 58 T-cell precursors were found in 2 × 10^4^ noncultured CD34^+^ CB cells, 13.4 × 10^3^ T-cell precursors were generated during a 7-day DL-4 culture (corresponding to a 230-fold increase [Table 1, third row]).

To identify the cell subset responsible for the marked increase in T-cell precursor frequency, serial dilutions of the distinct CD34^+^/CD7^−^, ETP, and proT1 DL-4 cell subsets from day 7 cultures were expanded in OP9/DL-1 cocultures, as described above. The CD34^+^/CD7^−^ cell population displayed much the T-lymphoid potential as untreated CD34^+^ cells (1 in 248). In contrast, ETPs and proT1 cells displayed much higher T-cell precursor frequencies (1 in 14.9 and 1 in 7.5, respectively) (Supporting Information Table S2). When injected into 4-week-old sublethally irradiated NSG mice, ETP and proT1 DL4 cells were able to reconstitute the thymus in three out of four mice as compared to none of the mice (0/2) injected with CD34^+^/CD7^−^ DL-4 cells (Supporting Information Fig. S2A). Interestingly, ETP and pro-T-cells retained the ability to produce B and myeloid cells (data not shown). This finding formally demonstrates that the increased T-lymphoid potential of DL-4 cells is correlated with their CD7 expression. Furthermore, mature CD4^+^/CD8^+^ double-positive cells and γδTCR^+^/CD3^+^ T cells emerged from ETPs and proT1 cells within 2 weeks, whereas an additional week was necessary to produce mature T cells from CD34^+^CD7^−^ DL-4 cells or uncultured CD34^+^ cells (Table 2). These results demonstrate that the ETP and proT1 progenitor subsets correspond to a more advanced T-cell developmental stage than the CD34^+^/CD7^−^ subset.

**Table 2:** The kinetics of T-cell differentiation for noncultured CD34^+^ cells and progenitor subsets retrieved from a DL-4 culture

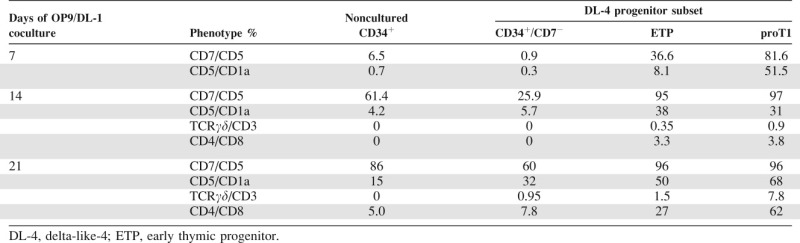

DL-4, delta-like-4; ETP, early thymic progenitor.

### DL-4 Progenitors Seed the Thymus and Give Rise to mature, Circulating T-Lymphocytes In Vivo

The T-cell potential of day-7 DL-4 cells was further investigated in two different xenotransplantation models. Four-week-old, sublethally irradiated NSG mice and newborn, nonirradiated NSG mice were injected (intravenously and intrahepatically, respectively) with either day-7 DL-4 cells (after exclusion of myeloid cells by FACS) or untreated CD34^+^ cells. Analyses were performed at 8 weeks or 4 weeks post-transplant, respectively.

**Figure 3:**
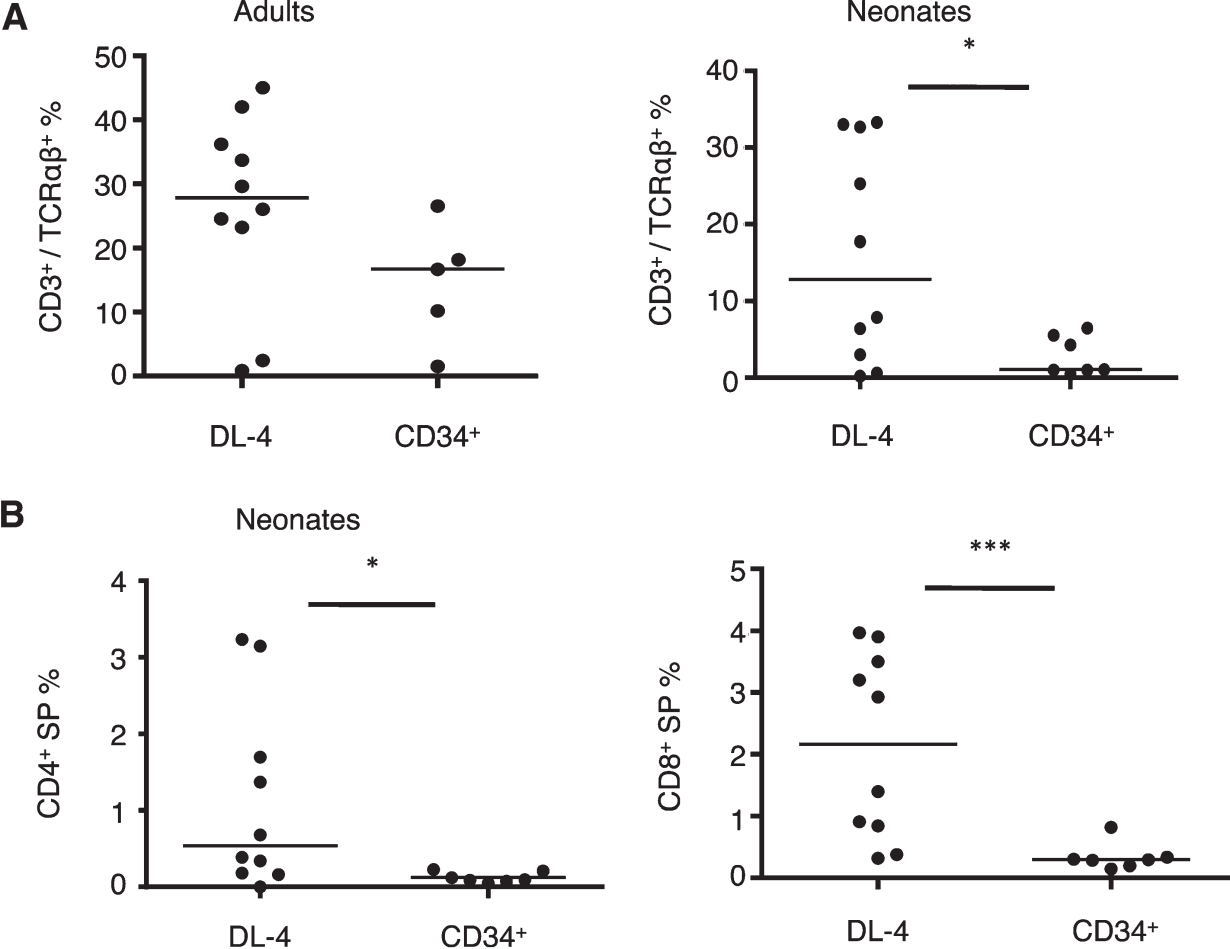
DL-4 cells can reconstitute the thymus in NOD/SCID/γc^−/−^ (NSG) mice. Human cord blood (CB) CD34^+^ cells were exposed to DL-4 in culture for 7 days. The DL-4-primed cells were sorted by flow cytometry, as described in Materials and Methods. Four-week-old irradiated NSG mice or nonirradiated newborn NSG mice were transplanted with 1.5 × 10^5^ noncultured CD34^+^ CB cells (adult: *n* = 7, newborn: *n* = 12) or 5 × 10^5^ sorted DL-4 cells (adult *n* = 12, newborn: *n* = 10) in three independent experiments. (A): Mice were sacrificed after 8 weeks and human CD45^+^ thymocytes were quantified by flow cytometry in each of the three groups. Positive thymic engraftment was defined as >2.5 × 10^3^ hCD45^+^ cells per thymus. Mice with positive thymic engraftment (adult: 10/12 in DL-4 vs. 6/7 in CD34^+^ group; newborn: 10/10 in DL-4 vs. 7/12 in CD34^+^ group) were further analyzed for CD4, CD8, CD3, and TCRαβ expression by gating on hCD45^+^/7AAD^−^ cells. The figure shows the percentage of CD3^+^/TCRαβ^+^ cells present in individual adult (left) and newborn (right) mice. Each point indicates a percentage observed in an individual mouse. Bars represent median values. *, *p* <.05. Data were combined from three independent experiments. In one of the untreated CD34^+^ recipients, CD3^+^/TCRαβ^+^ could not be analyzed due to technical reasons. (B): Percentage of CD4^+^ single-positive (SP) cells and CD8^+^ SP cells found in the thymus of individual newborn NSG mice. Horizontal bars represent median values, *, *p* <.05; ***, *p* <.001. Abbreviation: DL-4, delta-like-4.

In the adult cohort, we found thymic engraftment in 10 of the 12 mice injected with DL-4 cells, six of the seven mice injected with noncultured CD34^+^ cell, and none of the mice injected with cells from control-Fc cultures. In the nonirradiated newborn transplantation model, thymic engraftment was observed in all 10 DL-4 recipients but only seven of the 12 recipients of noncultured CD34^+^ cells. Intrathymic T-cell development was more advanced in recipients of DL-4-cells, as suggested by higher percentages of mature CD3^+^/TCRαβ^+^ cells, CD4 single-positive (SP) cells, and CD8 SP cells (Fig.3A, 3B and Supporting Information Fig. S2B). In nonirradiated newborn NSG mice, the low degree of BM chimerism (2%) contrasted with high thymic engraftment (38%) and suggested BM-independent repopulation of the thymus by DL-4 cells.

In the cohort of irradiated adult mice, mature T cells were detected in the spleen of four of the 12 DL-4 recipients but in only one of the seven noncultured CD34^+^ recipients. Furthermore, circulating mature T cells were found in the peripheral blood of three DL-4 recipients and none of the noncultured CD34^+^ cell recipients. These results demonstrate the DL-4 cells' ability to give rise to circulating mature T cells in vivo within 2 months. In both adult and newborn recipients, most of the thymic CD3^+^ T cells belonged to the αβ lineage (Supporting Information Fig. S2B) and displayed a polyclonal TCRβ VJ recombination pattern (Supporting Information Fig. S3).

### DL-4 Progenitors Seed the Thymus Faster than Untreated CD34^+^ Cells

To test the kinetic of thymic human engraftment and T-cell differentiation in vivo, we performed serial analysis in a large cohort of NSG neonates (*n* = 29) transplanted with DL-4 progenitors or untreated CD34^+^ cells. As depicted in Table 3, first signs of thymic engraftment with human cells could be detected in DL-4 recipients as soon as day 7 post-transplant. The majority of DL-4 recipients exhibited a robust ongoing intrathymic T-cell differentiation at day 14 (presence of intrathymic CD7^++^/CD5^+^/CD1a^+^ preT cells and in some case CD4^+^/CD3^+^ cells) (Supporting Information Fig. S2C). In contrast, thymopoiesis did not occur before day 21 post-transplant in recipients of untreated CD34^+^ cells (Table 3).

**Table 3:** Kinetics of thymic reconstitution after transfer of DL-4 cells or untreated CD34^+^ cells in nonirradiated newborn NOD/SCID/γc^−/−^ mice

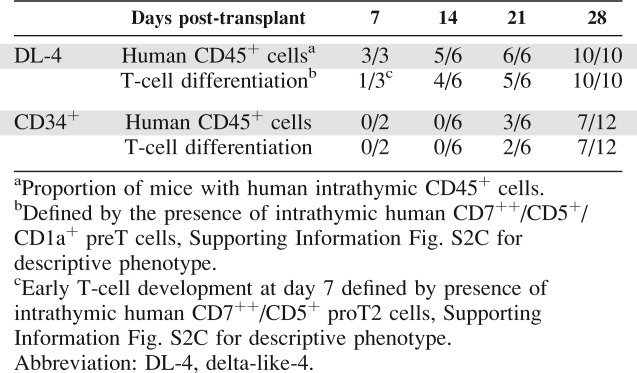

a Proportion of mice with human intrathymic CD45^+^ cells.

b Defined by the presence of intrathymic human CD7^++^/CD5^+^/CD1a^+^ preT cells, Supporting Information Fig. S2C for descriptive phenotype.

c Early T-cell development at day 7 defined by presence of intrathymic human CD7^++^/CD5^+^ proT2 cells, Supporting Information Fig. S2C for descriptive phenotype.

Abbreviation: DL-4, delta-like-4.

### Cotransplantation of DL-4 Progenitors and Untreated CD34^+^ Cells Promotes Thymopoiesis and Accelerates Peripheral T-Cell Reconstitution

**Figure 4:**
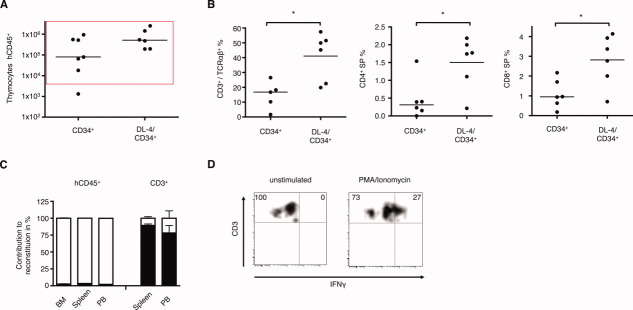
Cotransplantation of DL-4-primed progenitors and untreated CD34^+^ cells promotes thymopoiesis and accelerates peripheral T-cell reconstitution. (A): Four-week-old, sublethally irradiated NSG mice were coinjected with 5 × 10^5^ DL-4 cells (corresponding to the progeny of 7 × 10^4^ CD34^+^ cells) and 1.5 × 10^5^ noncultured CD34^+^ cells. Mice were sacrificed after 8 weeks and human CD45^+^ thymocytes were quantified by flow cytometry. Positive thymic engraftment was defined as >2.5 × 10^3^ hCD45^+^ cells per thymus (specimens within a red rectangle). Thymic reconstitution after cotransplantation was compared with that seen in mice injected with noncultured CD34^+^ cells alone. Horizontal bars indicate median values. (B): Analysis of intrathymic T-cell development in mice injected with noncultured CD34^+^ cells (*n* = 7, three independent experiments) or DL-4/CD34^+^ cells (*n* = 6, two independent experiments). The figure shows the percentage of CD3^+^/TCRαβ^+^ (left), CD4^+^ SP (middle), and CD8^+^ SP (right) cells found in individual NSG mice, with the median percentage indicated by horizontal bars. *, *p* <.05. In one of the untreated CD34^+^ recipients, CD3^+^/TCRαβ^+^ could not be analyzed due to technical reasons. (C): Four mice were coinjected with HLA-A2^−^ DL-4 cells and HLA-A2^+^ noncultured CD34^+^ cells. The relative contribution of the DL-4 cells and CD34^+^ cells to either CD45^+^ hematopoietic or CD3^+^ T-cell reconstitution was determined by quantifying HLA-A2^−^ cells (black bars) and HLA-A2^+^ cells (white bars) within a given population. Data are represented as the mean percentage ± SD. (D): Splenic T cells detected in cotransplanted mice were cultured with IL-2 and IL-7 for 7 days, then stimulated with PMA (50 ng/ml) and ionomycin (1 mg/ml) for 3 hours and assayed for intracytoplasmic IFN-γ expression. Abbreviations: BM, bone marrow; DL-4, delta-like-4; SP, single positive; PB, peripheral blood; PMA, phorbol mystirate acetate.

As described above, DL-4 cells were able to seed the thymus and give rise to mature circulating T cells when transferred into two different NSG mouse models of transplantation. Moreover, we found that T-cell reconstitution was faster in mice injected with DL-4 progenitors than in animals having received untreated CD34^+^ cell recipients. However, in the experiments presented above, mice received either sorted DL-4 cells or noncultured CD34^+^ cells. This transplantation condition differs markedly from a clinical CB transplantation setting, in which in vitro-generated, DL-4 cells would be injected in addition to untreated CD34^+^ cells. To further explore the in vivo T-lymphoid potential of DL-4 progenitors in a setting that more closely resembled clinical conditions, we cotransplanted 1.5 × 10^5^ untreated CD34^+^ cells and 5 × 10^5^ DL-4 progenitors from non-HLA-matched donors into 4-week-old NSG mice. Eight weeks after transplantation, we found thymic engraftment in all mice coinjected with DL-4/CD34^+^ cells. In the latter animals, levels of thymic engraftment were higher (Fig.4A) and intrathymic T-cell development was more advanced (Fig.4B) than in animals injected with untreated CD34^+^ cells. Two months after transplantation, we found mature T cells in the spleen of four of the 6 DL-4/CD34^+^ recipients, but only in four of the 12 DL-4 cell recipients and one of the seven mice injected with untreated CD34^+^ cells (Supporting Information Fig. S4A). Total intrasplenic T-cell counts (median [interquartile range]) in DL-4/CD34^+^ recipients were considerably higher than in DL-4 recipients (8.4 × 10^4^ [5.8 × 10^4^ – 4.4 × 10^5^] vs. 0.4 × 10^4^ [0.4–5.4 × 10^4^], respectively) and reached levels similar to those usually obtained at 12 weeks post-transplantation in CD34^+^ cell recipients (8.5 × 10^4^ [0.3–18.4]). We observed T cells in the peripheral blood of four of the six mice coinjected with DL-4/CD34^+^ cells. Upon polyclonal in vitro stimulation, T cells derived from spleens of DL-4/CD34^+^ reconstituted recipients were able to produced IFN-γ (Fig.4D).

Four recipients received DL-4 cells from an HLA-A2^-^ donor and untreated CD34^+^ cells from an HLA-A2^+^ donor. In this system, the engrafting cells’ expression (or not) of HLA-A2 identified them as being derived from DL-4 progenitors or untreated CD34^+^ cells. At the time of analysis, the vast majority (>97%) of engrafted human CD45^+^ cells in the BM, spleen, and peripheral blood were progeny of the untreated CD34^+^ cell fraction (Fig.4C and Supporting Information Fig. S4B). In contrast, 89% of splenic T cells and 78% of peripheral blood T cells were found to progeny of the DL-4 cell fraction (Fig.4C and Supporting Information Fig. S4B). Taken as a whole, these data show that the DL-4 cells' potential for promoting T-cell reconstitution is enhanced when they are cotransplanted with untreated CD34^+^ cells.

## DISCUSSION

In this study, we showed that brief exposure to immobilized DL-4 specifically induces a T-cell differentiation program in CB-derived CD34^+^ cells and thus enables the efficient generation of early T-cell progenitors in a feeder-cell-free culture system. There was strong evidence to suggest that DL-4 cells are indeed early T-cell progenitors, in view of their immunophenotype, gene expression pattern, TCR rearrangement pattern, and, most importantly, ability to accelerate T-cell differentiation both in vitro and in vivo. Strikingly, DL-4 cells adoptively transferred into NSG mice were able to repopulate the thymus, accelerate de novo thymopoiesis, give rise to mature peripheral T cells, and significantly quicken T-cell reconstitution.

In human thymopoiesis, ETP cells are the most immature T-cell-engaged population and account for less than 0.5% of the immature double-negative compartment [27–30]. We showed that 7 days of DL-4 culture particularly enabled the enrichment of this rare cell type. ETP and proT1 cells generated after 7-day culture did not express CD1a, had not undergone TCR rearrangements, and retained in vivo non-T-lymphoid potential, defining them as T-cell-engaged progenitors but not fully committed precursors. This is consistent with previous findings demonstrating that early stages of T-cell differentiation harbor plasticity for other lineages when placed in appropriate conditions [31]. Acquisition of the ETP and proT1 phenotypes was correlated with higher levels of the direct Notch targets *IL7Rα* and *PTα* [32–35] as well as of *RAG1*, which is essential for correct expression of a pre-TCR. Likewise, we found upregulation of BCL11B, which is involved in the survival and proliferation of T-cell precursors prior to β-selection [36–39]. The TCR rearrangement events found after long culture times were correlated with the appearance of proT1, proT2, and preT cells. These three cells populations had much the same TCR patterns as we (unpublished data) and others have observed for native thymopoiesis [32].

Induction of CD7 expression within the ETP and proT1-like DL-4 progenitor cells correlated with greater in vitro and in vivo T-cell potential. Furthermore, ETP and proT1 cells sorted from day 7 DL-4 cultures were able to complete in vitro T-cell differentiation (in secondary OP9/DL-1 coculture) to a higher extent than CD34^+^/CD7^−^ DL-4 cells or untreated CB CD34^+^ cells did. This observation provides further functional evidence of an advanced T-cell development stage. Taken as a whole, these in vitro data clearly show that the DL-4 culture described herein recapitulate the early T-cell development stages of native thymopoiesis (as has already been described in OP9/DL-1 cocultures [12, 13]).

At the pre-β-selection stages, the T-cell differentiation of DL-4-progenitors followed the same differentiation pattern as reported in the literature for OP9/DL-1 cocultures [12, 33] and in our own unpublished observations. In contrast to OP9-DL-1 cocultures (which support the generation of mature T cells in vitro), the T-cell differentiation induced by DL-4 is blocked at the preT cell stage. This agrees with previous demonstrations in which signals other than Notch1 activation (i.e., stimulation of CXCR4 by SDF1a and activation of the Wnt pathway) are required to successfully pass through the β-selection checkpoint [34, 35]. These signals are partially secreted/expressed by OP9/DL-1 cells [40, 41]. Stimulation of CXCR4 and Wnt signaling in a murine DL-4 culture was found to partially overcome the pre-β selection block [34].

This particularity of immobilized Notch-ligand culture systems probably also explains the emergence of an NK-like population in the DL-4 culture. T and NK cells share early, Notch-dependent differentiation steps [42]. Although the CD7^++^ progenitors observed in both OP9/DL-1 coculture and native thymopoiesis have T/NK potential in vitro, the emergence of a substantial NK-subset is found in neither OP9/DL-1 nor the normal thymus, possibly due to the presence of further biological signals [12, 13, 41, 42]. Given the absence of such signals (i.e., Wnt signaling and CXCR4 signaling) in DL-4 culture, we consider that the NK cells emerged from the CD7^++^ bipotent T/NK progenitor via a biased differentiation pathway. The occurrence of NK cells in feeder-cell-free Notch-ligand cultures has been reported and was attributed to a lack of Wnt signaling [17, 41, 42]. In our opinion, the occurrence of biased NK cells after prolonged DL-4 culture (i.e., ≥10 days) provides a further argument for using day 7 DL-4 cells culture in transplantation experiments.

Long-term DL-1 culture (i.e., for more than 2 weeks) enables the relatively specific in vitro expansion of CD34^+^ CB cells [20]. This contrasts markedly with DL-4 culture in which CD34^+^ expression decreases after 1 week. ETP, ProT1, and NK-biased CD56^+^ cells also appear in DL-1 cultures, albeit at lower frequencies and at later culture time points. These findings indicate that DL-1 and DL-4 differ in their ability to induce the generation of immature hematopoietic cells and T-cell-engaged cells, respectively. Recent findings provide a partial biological explanation for this difference. It was recently found that the DL-1-dependent expansion of CD34^+^ hematopoietic progenitors was mediated by Notch-2 [43]. In contrast, T-cell development is strictly dependent on Notch-1 signaling [19, 20]. Furthermore, DL-4 was unambiguously found to be the essential Notch1 ligand in thymopoiesis and more potent than DL-1 for inducing the T-cell differentiation program [22, 44, 45]. These arguments sustained our choice to use DL-4 and a short culture period for this protocol.

The ability of adoptively transferred, in vitro-generated murine T-cell precursors to promote T-cell reconstitution in an allotransplant model has already been unambiguously demonstrated [15, 16]. Likewise, human T-cell progenitors generated from CB cells or mobilized HSCs on feeder cells expressing Notch ligands can transiently promote and accelerate thymopoiesis in humanized mice [12, 13]. When human CD34^+^ cells were expanded on plate-bound DL-1, only the injection of very high cell numbers was capable of achieving thymic engraftment in NSG mice [17]. However, none of these studies described positive effects on peripheral T-cell reconstitution [12, 13, 17].

Our present results demonstrate that DL-4-primed cells displayed greater thymopoietic potential after adoptive transfer into NSG mice. Intrathymic T-cell differentiation was faster in DL-4 injected mice than in mice injected with noncultured CD34^+^ cells. Importantly, T cells derived from injected, DL-4-exposed T-cell progenitors displayed a conventional and polyclonal CD4, CD8, and TCRαβ profile.

T-cell progenitors generated during DL-4 culture accelerated thymopoiesis to the same extent as OP9-DL-1-generated T-cell progenitors did (data not shown). Furthermore, we detected a higher degree of peripheral T-cell reconstitution in DL-4 recipients 2 months after transplantation; this constitutes a truly novel feature of the DL-4 expansion. Thymopoiesis and peripheral T-cell reconstitution were still further improved when DL-4 cells were coinjected with untreated CD34^+^ cells. The DL-4 cells had selectively repopulated the T-cell compartment by 2 months post-transplantation, whereas the untreated CD34^+^ cells had almost exclusively reconstituted the other hematopoietic compartments. The clearly improved T-cell recovery after cotransplantation highlights the synergy between the DL-4 primed unit and the untreated CB unit. We presume that (a) DL-4 T-cell progenitors instantly provide a wave of thymopoiesis and T-cell regeneration and (b) long-term T-cell development is sustained by the untreated CB unit, which can sustain T-cell progenitors generation in the recipient's bone marrow.

Just 1 week of DL-4 culture generated enough T-cell progenitors to envisage their adoptive transfer in vivo. In contrast to OP9-DL-1 system, the proposed DL-4 system does not contain genetically modified murine cells and thus circumvents a considerable safety risk. The use of magnetically sorted CD34^+^ cells (as routinely performed in haploidentical human transplantation settings), with no enrichment of the more immature subsets (i.e., CD34^+^/CD38^−^) resolves a further obstacle to human application. The cotransplantation design mimics HLA-mismatched dUCBT quite closely and suggest a potential clinical application. In such a situation, DL-4 culture could be initiated with a single CB unit during 7 days, with infusion of the culture product at the same time as (or shortly after) transplantation of the untreated CB unit. In this straightforward instance, the use of DL-4 T-cell progenitors would advantageously bypass the need for de novo T-cell progenitor generation in the patient's BM; this would quicken immune reconstitution after HSCT and should reduce post-transplantation morbidity and mortality due to infections.

In addition to this direct therapeutic approach, other diseases characterized by impairments of early intrathymic T-cell development and alterations in thymic structure could potentially be treated with DL-4-primed progenitors. The latter are capable of inducing thymopoiesis even in the highly disturbed thymic microenvironment of irradiated, immunodeficient mice. In severe combined immunodeficiencies, the absence of a physiological interaction between thymic progenitors and the thymic stroma means that thymopoiesis is still disturbed after HSCT [46, 47]. Likewise, thymopoiesis is affected in chronic inflammatory disorders, HIV infection, and in the aftermath of cytotoxic, pretransplantation conditioning [8, 48–50]. Given the DL-4 primed progenitors' potential to bypass the initial stages in thymic T-cell differentiation, one can legitimately hypothesize that patients affected by these conditions would benefit from treatment with in vitro-generated progenitors. The presence of a great number of functional T-cell progenitors could provide a physiological stimulus to the altered thymic stroma and thereby help regenerate thymic function and organization.

## CONCLUSIONS

This study first described a feeder-cell-free Notch-ligand culture system specifically for the rapid generation of human T-cell progenitors in vitro. Moreover, we provide the first evidence that human in vitro-generated T-cell progenitors can further differentiate into mature, polyclonal, functional peripheral T-cells in the perturbed thymic microenvironment of the NSG mouse. Coinjection of DL-4-primed and untreated CD34^+^ CB cells further improved T-cell recovery under experimental conditions that quite closely mimic the clinical setting of dUCBT. This provides a strong argument for the therapeutic potential of DL-4 cells.

## Notes

Fig S1 DL‐4 cells upregulate CD45RA and CXCR4 during culture A: Phenotypic analysis of CD34 and CD7 expression by DL‐4 cells harvested after 3, 7 and 14 days of culture (upper line). The surface expression of CD45RA (middle line) and CXCR4 (lower line) was tested within the CD34+/CD7−, CD34+/CD7+ (ETP) and CD34−/CD7++ (proT) subsets. RCN: relative cell number. B: Flow cytometric analysis of surface CXCR4 expression, as measured by gating on CD34− /CD7++, CD34−/CD7++/CD5+ and CD34−/CD7++/CD56+ subsets.

Fig S1 DL‐4 cells upregulate CD45RA and CXCR4 during culture A: Phenotypic analysis of CD34 and CD7 expression by DL‐4 cells harvested after 3, 7 and 14 days of culture (upper line). The surface expression of CD45RA (middle line) and CXCR4 (lower line) was tested within the CD34+/CD7−, CD34+/CD7+ (ETP) and CD34−/CD7++ (proT) subsets. RCN: relative cell number. B: Flow cytometric analysis of surface CXCR4 expression, as measured by gating on CD34− /CD7++, CD34−/CD7++/CD5+ and CD34−/CD7++/CD56+ subsets.

Fig S2 DL‐4 cells can reconstitute the thymus when transferred into irradiated adult NOD/SCID/γc−/− (NSG) mice and into non‐irradiated, newborn NSG mice and accelerates thymopoesis in vivo A: CD34+/CD7− and ETP /proT1 cells sorted from a 7‐day DL‐4 culture were transplanted into 4 week old irradiated NSG. Thymic reconstitution was assessed after 8 weeks by flow cytometry. CD4, CD8, CD3 and TCRαβ expression was studied within hCD45+/7AAD‐ cells. B: Irradiated adult NSG mice (upper group) and non‐irradiated newborn NSG mice (lower group) were injected with 5 × 105 sorted DL‐4 progenitors or 1.5 × 105 non‐cultured CD34+ cells. Thymus reconstitution was assessed 8 weeks (in adult recipients) or 4 weeks (in newborn recipients) after transplantation. The figure shows the flow cytometry analysis for thymic reconstitution as described above. C: A total of 29 non‐irradiated newborn NSG were transplanted with 5 × 105 sorted DL‐4 progenitors or 1.5 × 105 non‐cultured CD34+ cells in two independent transplantation series. Results of the thymic reconstitution kinetics in DL‐4 versus untreated CD34+ cells are described in Table 4. Phenotypic images of representative thymi recovered from DL‐4 recipients at 7 and 14 days post‐transplant are shown.

Fig S2 DL‐4 cells can reconstitute the thymus when transferred into irradiated adult NOD/SCID/γc−/− (NSG) mice and into non‐irradiated, newborn NSG mice and accelerates thymopoesis in vivo A: CD34+/CD7− and ETP /proT1 cells sorted from a 7‐day DL‐4 culture were transplanted into 4 week old irradiated NSG. Thymic reconstitution was assessed after 8 weeks by flow cytometry. CD4, CD8, CD3 and TCRαβ expression was studied within hCD45+/7AAD‐ cells. B: Irradiated adult NSG mice (upper group) and non‐irradiated newborn NSG mice (lower group) were injected with 5 × 105 sorted DL‐4 progenitors or 1.5 × 105 non‐cultured CD34+ cells. Thymus reconstitution was assessed 8 weeks (in adult recipients) or 4 weeks (in newborn recipients) after transplantation. The figure shows the flow cytometry analysis for thymic reconstitution as described above. C: A total of 29 non‐irradiated newborn NSG were transplanted with 5 × 105 sorted DL‐4 progenitors or 1.5 × 105 non‐cultured CD34+ cells in two independent transplantation series. Results of the thymic reconstitution kinetics in DL‐4 versus untreated CD34+ cells are described in Table 4. Phenotypic images of representative thymi recovered from DL‐4 recipients at 7 and 14 days post‐transplant are shown.

Fig S2 DL‐4 cells can reconstitute the thymus when transferred into irradiated adult NOD/SCID/γc−/− (NSG) mice and into non‐irradiated, newborn NSG mice and accelerates thymopoesis in vivo A: CD34+/CD7− and ETP /proT1 cells sorted from a 7‐day DL‐4 culture were transplanted into 4 week old irradiated NSG. Thymic reconstitution was assessed after 8 weeks by flow cytometry. CD4, CD8, CD3 and TCRαβ expression was studied within hCD45+/7AAD‐ cells. B: Irradiated adult NSG mice (upper group) and non‐irradiated newborn NSG mice (lower group) were injected with 5 × 105 sorted DL‐4 progenitors or 1.5 × 105 non‐cultured CD34+ cells. Thymus reconstitution was assessed 8 weeks (in adult recipients) or 4 weeks (in newborn recipients) after transplantation. The figure shows the flow cytometry analysis for thymic reconstitution as described above. C: A total of 29 non‐irradiated newborn NSG were transplanted with 5 × 105 sorted DL‐4 progenitors or 1.5 × 105 non‐cultured CD34+ cells in two independent transplantation series. Results of the thymic reconstitution kinetics in DL‐4 versus untreated CD34+ cells are described in Table 4. Phenotypic images of representative thymi recovered from DL‐4 recipients at 7 and 14 days post‐transplant are shown.

Figure S3 DL‐4 cells give rise to polyclonal thymocytes in recipient mice TCRβ VJ rearrangement patterns were analyzed for a number of reconstituted thymuses from adult and newborn NSG recipients. A) The TCRβ VJ recombination pattern in a representative thymus reconstituted with DL‐4 cells. The image represents the result of an ImmunTraCkeR® Multiplex PCR that detects all recombination events between the TCRβ V and TCRβ J loci. B) The diagram shows the combinatorial diversity of the TCRβ VJ rearrangements in reconstituted thymuses from mice injected with DL‐4‐cells (left column), non‐cultured CD34+ cells (middle column) and control T lymphocytes from healthy donors (right column). Each dot indicates the combinatorial diversity of an individual sample. Bars indicate median values.

Figure S4 DL‐4 cells give rise to peripheral T‐cells in recipient mice and specifically reconstitute the T‐cell compartment A) Mature T‐cells were found in the spleen of 4 out of 12 DL‐4 cell recipients, 1 out of 7 noncultured CD34+ cell recipient and 4 out of 6 mice co‐transplanted with DL‐4 and CD34+ cells. The Figure shows a flow cytometry analysis of CD3, CD4 and TCRαβ expression in a representative spleen from each type of transplantation. B) Mice were co‐injected with HLA‐A2‐ DL‐4 cells and HLA‐A2+ untreated CD34+ CB cells. The upper panel shows an experiment gated on hCD45+ spleen cells. HLA‐A2 expression was examined by gating on the entire hCD45+ spleen cell population (left histogram) and on the splenic CD3+/TCRαβ+ T‐cells in particular (right histogram). The flow cytometry analysis pictured is representative of three different mice.
