# Supplementary figures and images for: Human T-Lymphoid Progenitors Generated in a Feeder-Cell-Free Delta-Like-4 Culture System Promote T-Cell Reconstitution in NOD/SCID/γc−/− Mice
Source: Stem Cells. 2012 Jul 24;30:1771–80. doi: 10.1002/stem.1145 (PMC3531890; doi:10.1002/stem.1145)

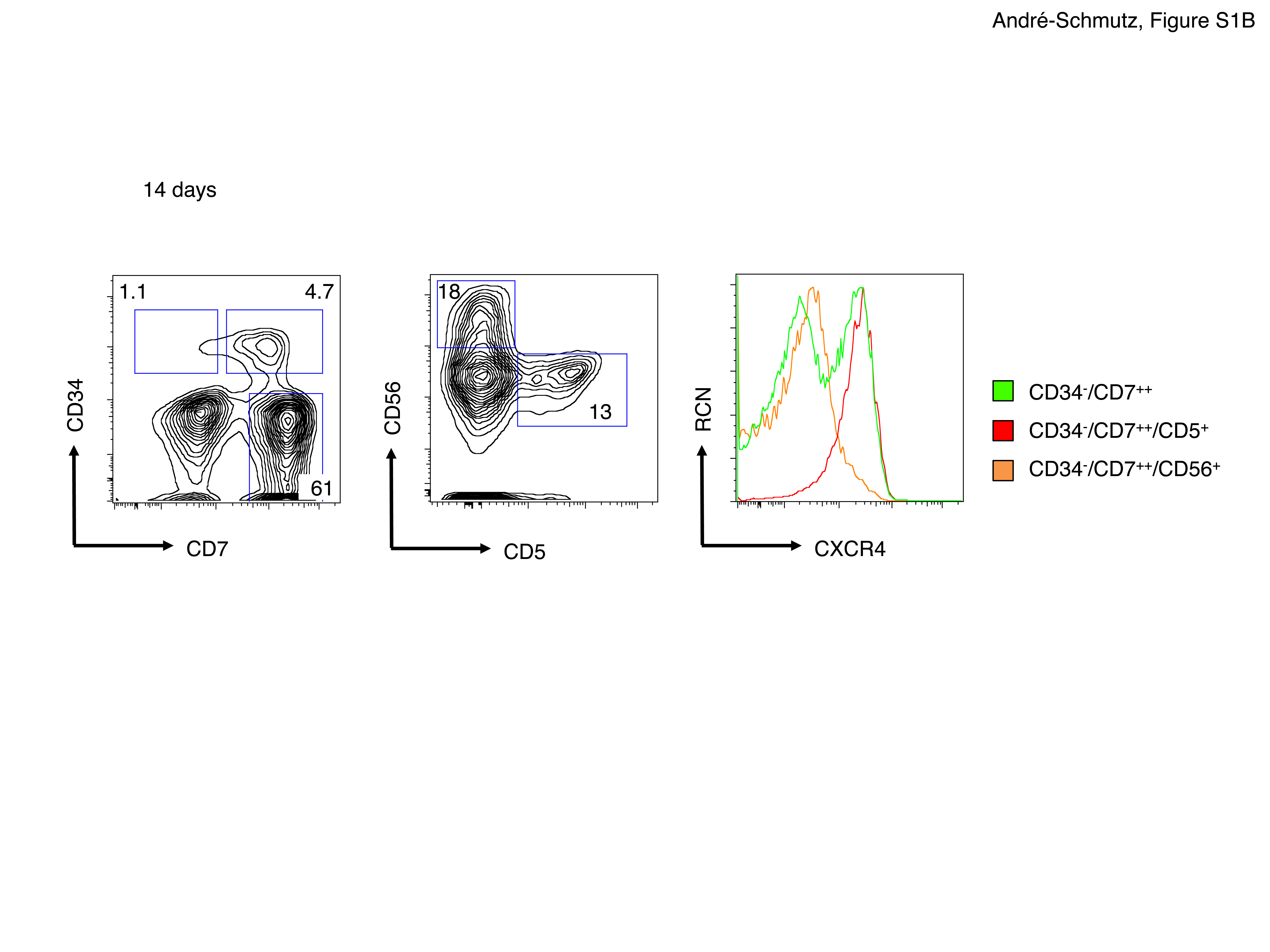

Supplement: Fig S1 — DL‐4 cells upregulate CD45RA and CXCR4 during culture A: Phenotypic analysis of CD34 and CD7 expression by DL‐4 cells harvested after 3, 7 and 14 days of culture (upper line). The surface expression of CD45RA (middle line) and CXCR4 (lower line) was tested within the CD34+/CD7−, CD34+/CD7+ (ETP) and CD34−/CD7++ (proT) subsets. RCN: relative cell number. B: Flow cytometric analysis of surface CXCR4 expression, as measured by gating on CD34− /CD7++, CD34−/CD7++/CD5+ and CD34−/CD7++/CD56+ subsets. [file stem0030-1771-SD1.tif]

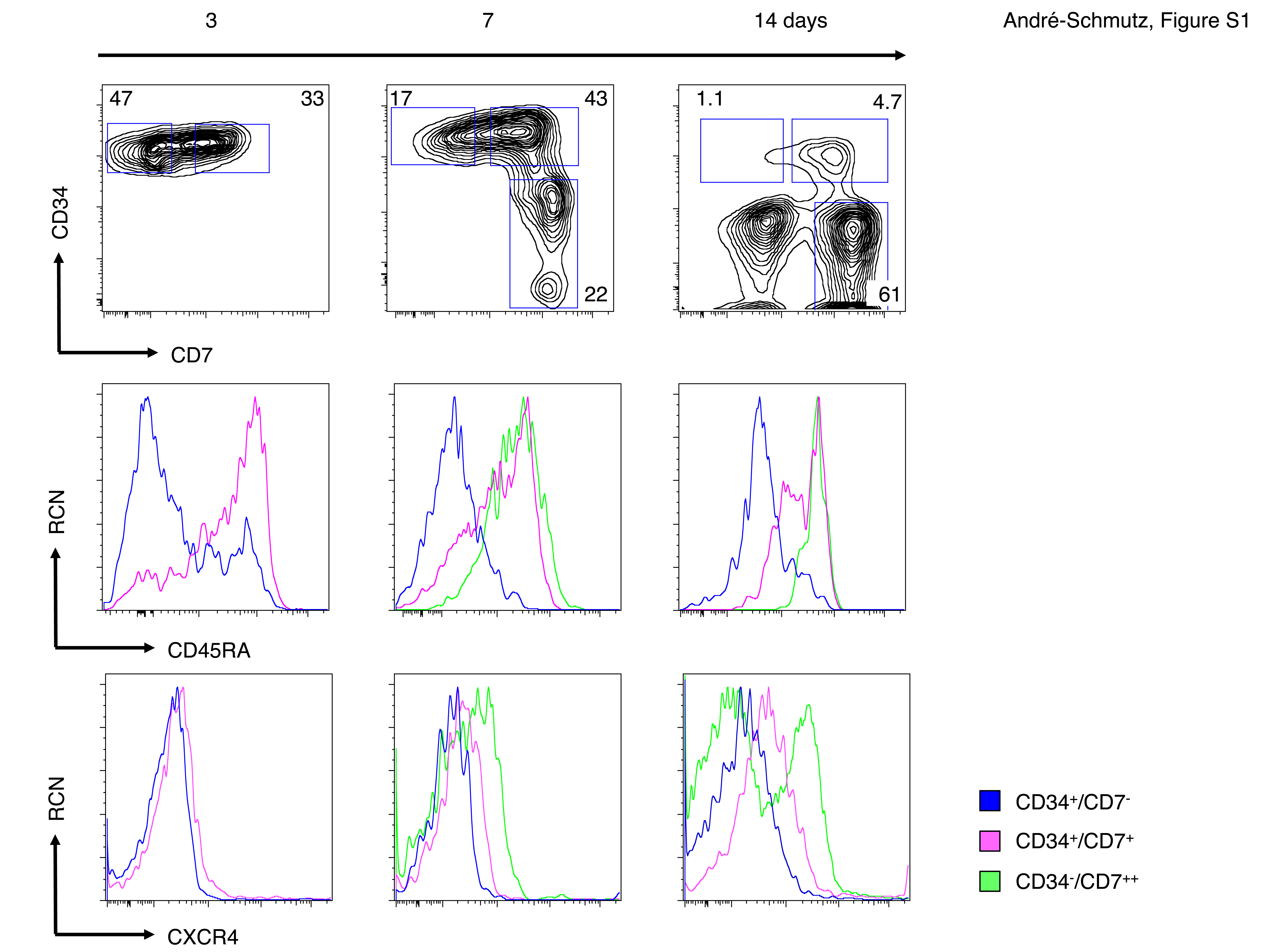

Supplement: Fig S1 — DL‐4 cells upregulate CD45RA and CXCR4 during culture A: Phenotypic analysis of CD34 and CD7 expression by DL‐4 cells harvested after 3, 7 and 14 days of culture (upper line). The surface expression of CD45RA (middle line) and CXCR4 (lower line) was tested within the CD34+/CD7−, CD34+/CD7+ (ETP) and CD34−/CD7++ (proT) subsets. RCN: relative cell number. B: Flow cytometric analysis of surface CXCR4 expression, as measured by gating on CD34− /CD7++, CD34−/CD7++/CD5+ and CD34−/CD7++/CD56+ subsets. [file stem0030-1771-SD2.tif]

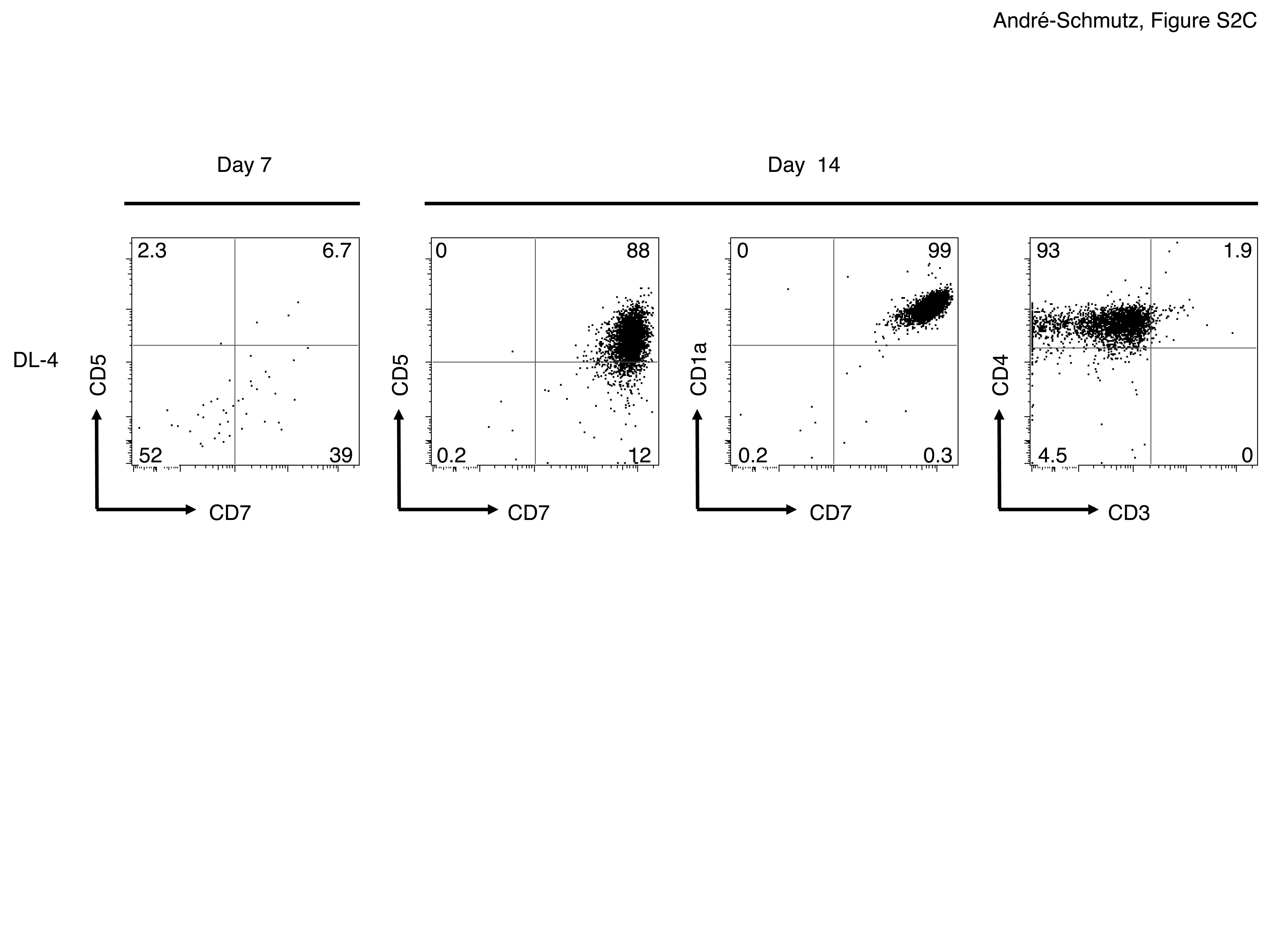

Supplement: Fig S2 — DL‐4 cells can reconstitute the thymus when transferred into irradiated adult NOD/SCID/γc−/− (NSG) mice and into non‐irradiated, newborn NSG mice and accelerates thymopoesis in vivo A: CD34+/CD7− and ETP /proT1 cells sorted from a 7‐day DL‐4 culture were transplanted into 4 week old irradiated NSG. Thymic reconstitution was assessed after 8 weeks by flow cytometry. CD4, CD8, CD3 and TCRαβ expression was studied within hCD45+/7AAD‐ cells. B: Irradiated adult NSG mice (upper group) and non‐irradiated newborn NSG mice (lower group) were injected with 5 × 10^5^ sorted DL‐4 progenitors or 1.5 × 10^5^ non‐cultured CD34+ cells. Thymus reconstitution was assessed 8 weeks (in adult recipients) or 4 weeks (in newborn recipients) after transplantation. The figure shows the flow cytometry analysis for thymic reconstitution as described above. C: A total of 29 non‐irradiated newborn NSG were transplanted with 5 × 10^5^ sorted DL‐4 progenitors or 1.5 × 10^5^ non‐cultured CD34+ cells in two independent transplantation series. Results of the thymic reconstitution kinetics in DL‐4 versus untreated CD34+ cells are described in Table 4. Phenotypic images of representative thymi recovered from DL‐4 recipients at 7 and 14 days post‐transplant are shown. [file stem0030-1771-SD3.tif]

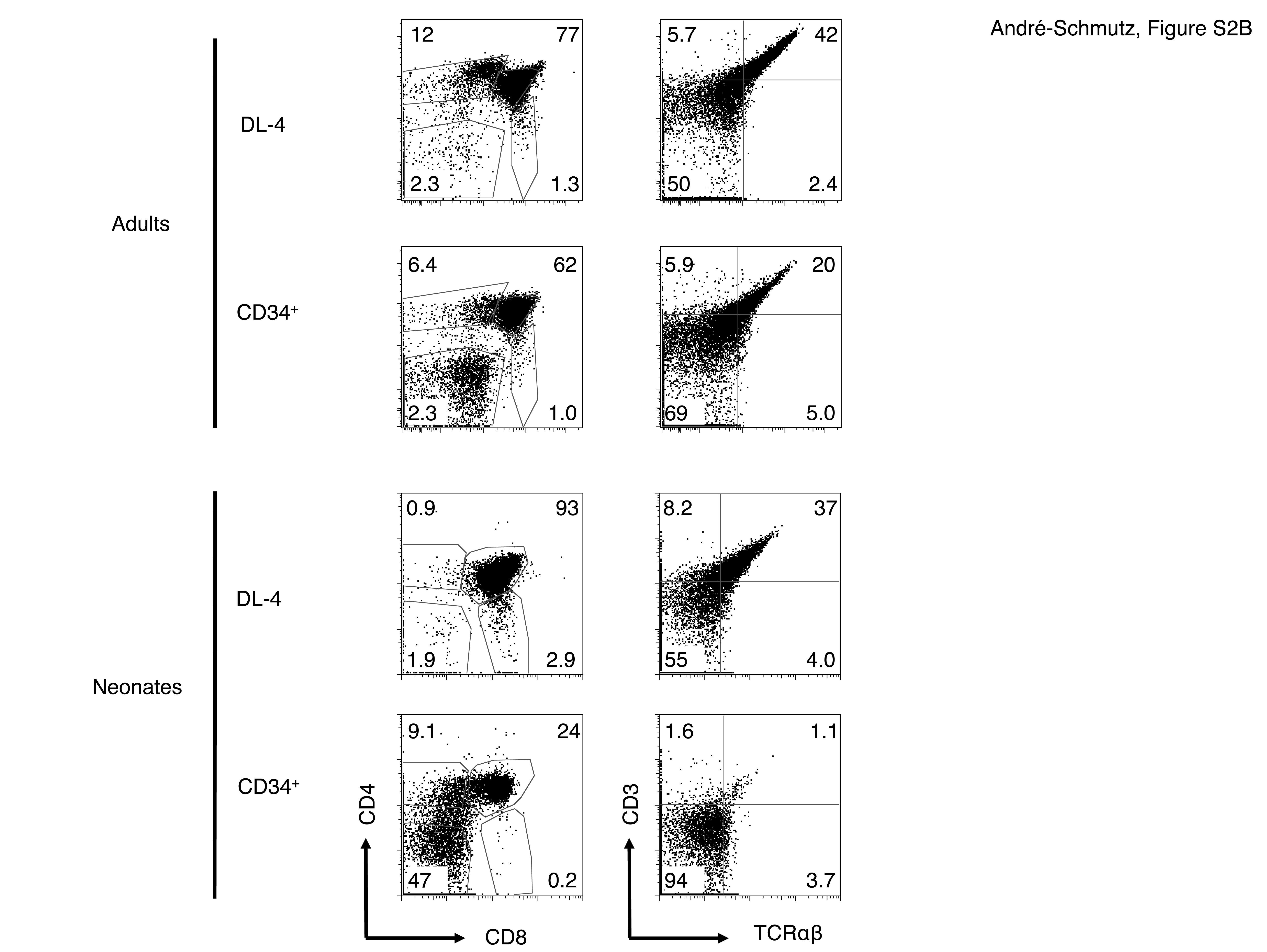

Supplement: Fig S2 — DL‐4 cells can reconstitute the thymus when transferred into irradiated adult NOD/SCID/γc−/− (NSG) mice and into non‐irradiated, newborn NSG mice and accelerates thymopoesis in vivo A: CD34+/CD7− and ETP /proT1 cells sorted from a 7‐day DL‐4 culture were transplanted into 4 week old irradiated NSG. Thymic reconstitution was assessed after 8 weeks by flow cytometry. CD4, CD8, CD3 and TCRαβ expression was studied within hCD45+/7AAD‐ cells. B: Irradiated adult NSG mice (upper group) and non‐irradiated newborn NSG mice (lower group) were injected with 5 × 10^5^ sorted DL‐4 progenitors or 1.5 × 10^5^ non‐cultured CD34+ cells. Thymus reconstitution was assessed 8 weeks (in adult recipients) or 4 weeks (in newborn recipients) after transplantation. The figure shows the flow cytometry analysis for thymic reconstitution as described above. C: A total of 29 non‐irradiated newborn NSG were transplanted with 5 × 10^5^ sorted DL‐4 progenitors or 1.5 × 10^5^ non‐cultured CD34+ cells in two independent transplantation series. Results of the thymic reconstitution kinetics in DL‐4 versus untreated CD34+ cells are described in Table 4. Phenotypic images of representative thymi recovered from DL‐4 recipients at 7 and 14 days post‐transplant are shown. [file stem0030-1771-SD4.tif]

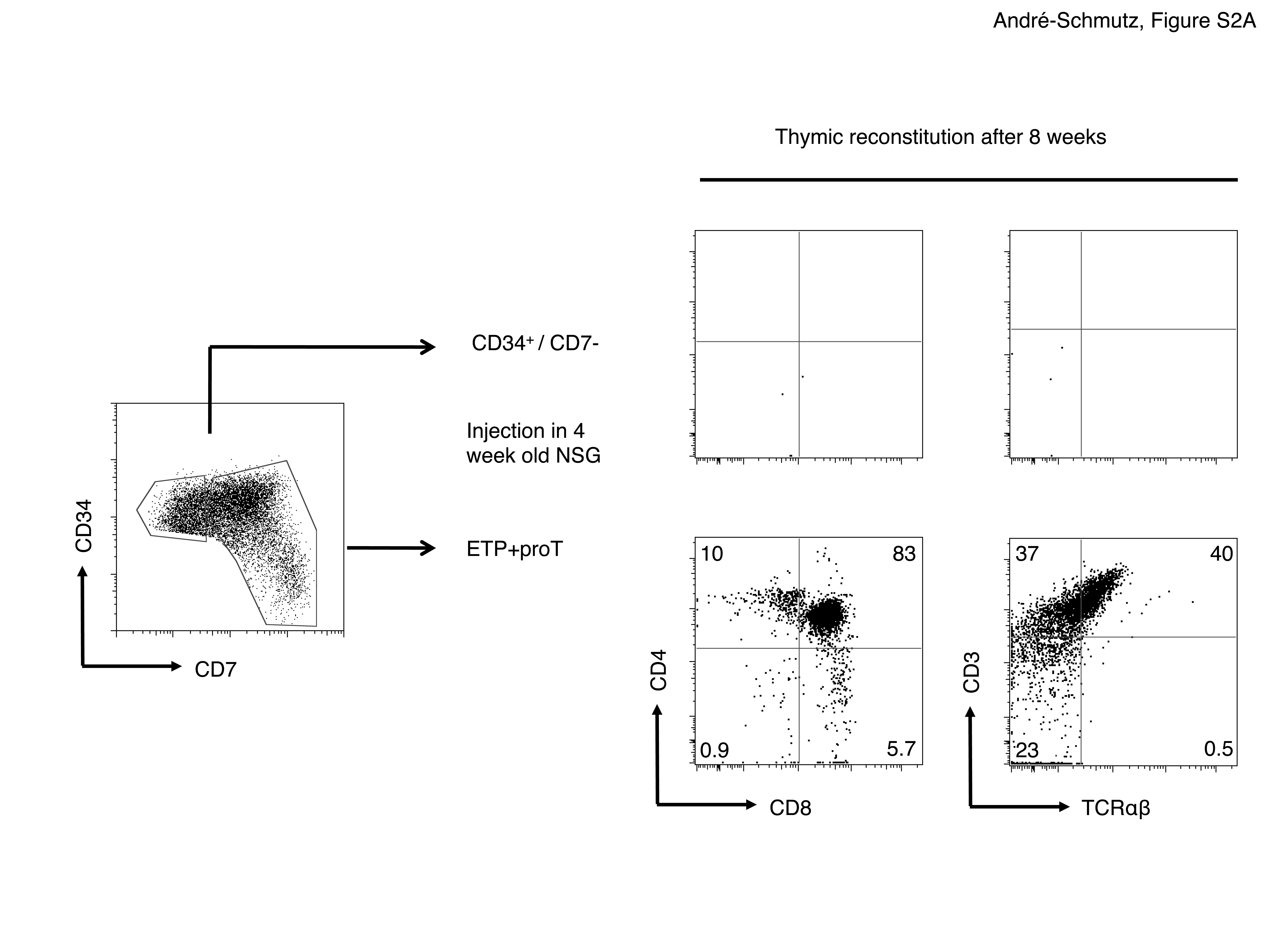

Supplement: Fig S2 — DL‐4 cells can reconstitute the thymus when transferred into irradiated adult NOD/SCID/γc−/− (NSG) mice and into non‐irradiated, newborn NSG mice and accelerates thymopoesis in vivo A: CD34+/CD7− and ETP /proT1 cells sorted from a 7‐day DL‐4 culture were transplanted into 4 week old irradiated NSG. Thymic reconstitution was assessed after 8 weeks by flow cytometry. CD4, CD8, CD3 and TCRαβ expression was studied within hCD45+/7AAD‐ cells. B: Irradiated adult NSG mice (upper group) and non‐irradiated newborn NSG mice (lower group) were injected with 5 × 10^5^ sorted DL‐4 progenitors or 1.5 × 10^5^ non‐cultured CD34+ cells. Thymus reconstitution was assessed 8 weeks (in adult recipients) or 4 weeks (in newborn recipients) after transplantation. The figure shows the flow cytometry analysis for thymic reconstitution as described above. C: A total of 29 non‐irradiated newborn NSG were transplanted with 5 × 10^5^ sorted DL‐4 progenitors or 1.5 × 10^5^ non‐cultured CD34+ cells in two independent transplantation series. Results of the thymic reconstitution kinetics in DL‐4 versus untreated CD34+ cells are described in Table 4. Phenotypic images of representative thymi recovered from DL‐4 recipients at 7 and 14 days post‐transplant are shown. [file stem0030-1771-SD5.tif]

**A**

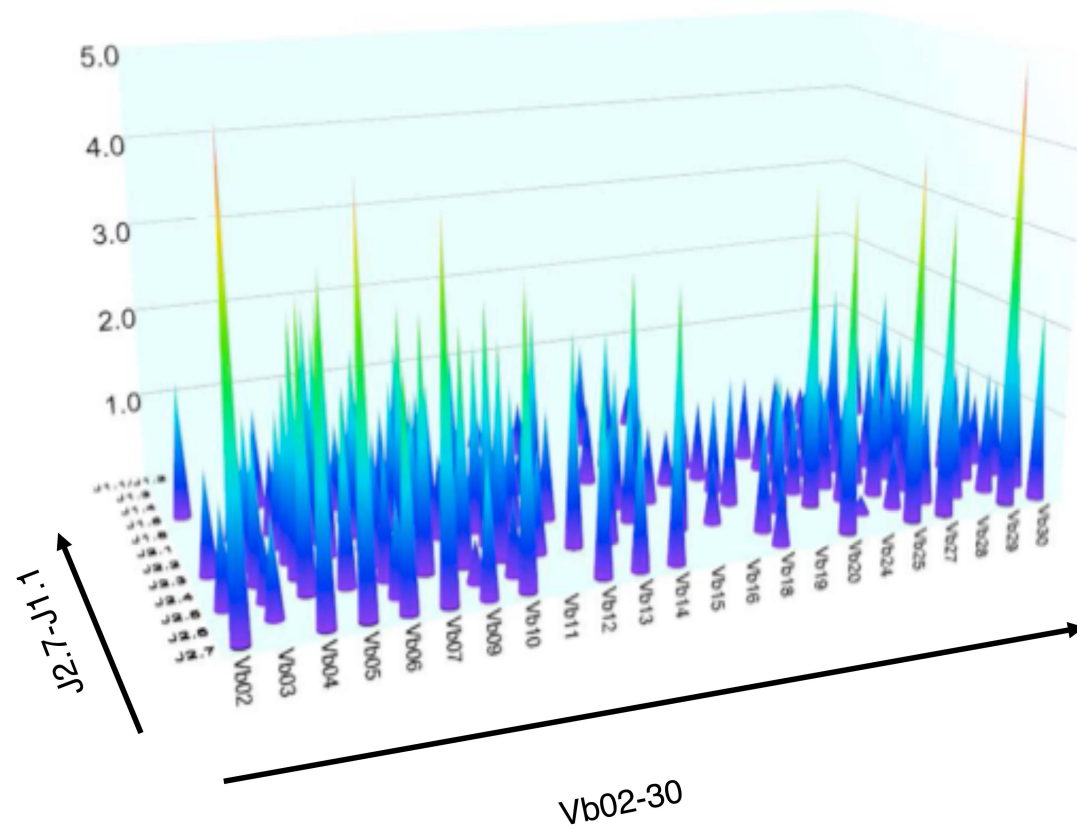

**B**

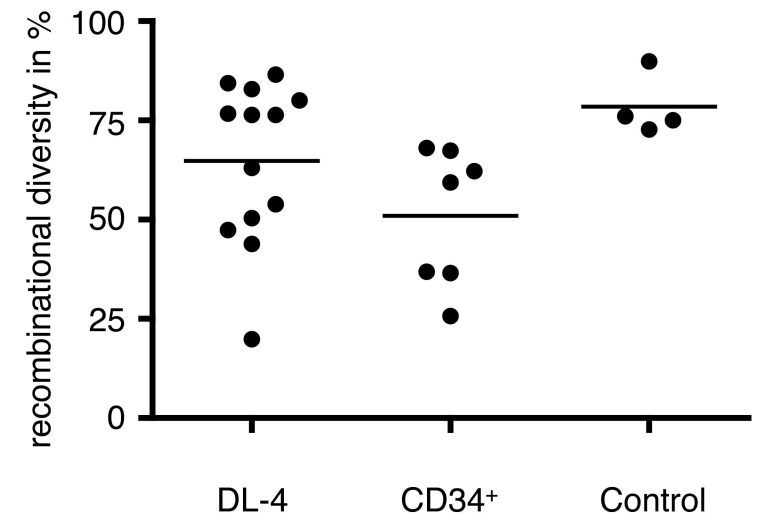

Supplement: Figure S3 — DL‐4 cells give rise to polyclonal thymocytes in recipient mice TCRβ VJ rearrangement patterns were analyzed for a number of reconstituted thymuses from adult and newborn NSG recipients. A) The TCRβ VJ recombination pattern in a representative thymus reconstituted with DL‐4 cells. The image represents the result of an ImmunTraCkeR® Multiplex PCR that detects all recombination events between the TCRβ V and TCRβ J loci. B) The diagram shows the combinatorial diversity of the TCRβ VJ rearrangements in reconstituted thymuses from mice injected with DL‐4‐cells (left column), non‐cultured CD34+ cells (middle column) and control T lymphocytes from healthy donors (right column). Each dot indicates the combinatorial diversity of an individual sample. Bars indicate median values. [file stem0030-1771-SD6.pdf]

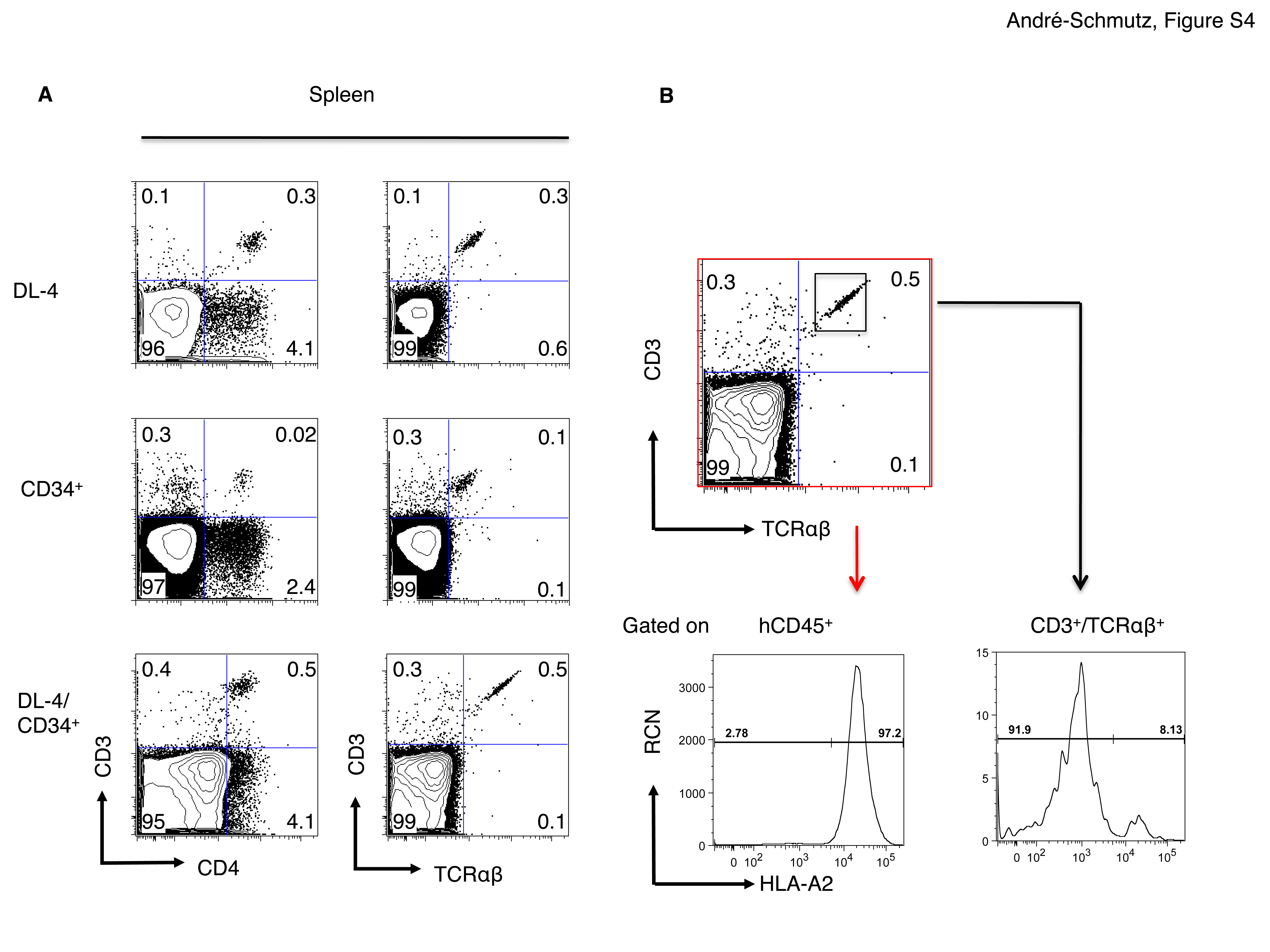

Supplement: Figure S4 — DL‐4 cells give rise to peripheral T‐cells in recipient mice and specifically reconstitute the T‐cell compartment A) Mature T‐cells were found in the spleen of 4 out of 12 DL‐4 cell recipients, 1 out of 7 noncultured CD34+ cell recipient and 4 out of 6 mice co‐transplanted with DL‐4 and CD34+ cells. The Figure shows a flow cytometry analysis of CD3, CD4 and TCRαβ expression in a representative spleen from each type of transplantation. B) Mice were co‐injected with HLA‐A2‐ DL‐4 cells and HLA‐A2+ untreated CD34+ CB cells. The upper panel shows an experiment gated on hCD45+ spleen cells. HLA‐A2 expression was examined by gating on the entire hCD45+ spleen cell population (left histogram) and on the splenic CD3+/TCRαβ+ T‐cells in particular (right histogram). The flow cytometry analysis pictured is representative of three different mice. [file stem0030-1771-SD7.tif]
