# Supplementary material for: Human T-Lymphoid Progenitors Generated in a Feeder-Cell-Free Delta-Like-4 Culture System Promote T-Cell Reconstitution in NOD/SCID/γc−/− Mice
Source: Stem Cells. 2012 Jul 24;30:1771–80. doi: 10.1002/stem.1145 (PMC3531890; doi:10.1002/stem.1145)
Supplement: Supplementary Table 1 [file stem0030-1771-SD8.pdf]

Table S1

	Culture time in days				
TCR rearrangement event	0	7	10	14	18
Dδ2-Dδ3 and Vδ2-Dδ3	-	-	+	+	+
Dδ2-Jδ1	-	-	+	+	+
Vd1-Jd1	-	-	-	+	+
Vγ9 and Vγ11	-	-	-	+	+
Dβ1-Jβ	-	-	-	+	+
Vβ-Jβ2	-	-	-	-	+

Table S1: Kinetics of TCR rearrangement events during DL-4 culture
