# Supplementary material for: Human T-Lymphoid Progenitors Generated in a Feeder-Cell-Free Delta-Like-4 Culture System Promote T-Cell Reconstitution in NOD/SCID/γc−/− Mice
Source: Stem Cells. 2012 Jul 24;30:1771–80. doi: 10.1002/stem.1145 (PMC3531890; doi:10.1002/stem.1145)
Supplement: Supplementary Table 2 [file stem0030-1771-SD9.pdf]

Table S2

**Progenitor subset**[a]	**T cell precursor frequency**$^{-1}$ [b]
**CD34^{+}/CD7^{-}**	**248** [191-305]
**ETP**	**14.9** [12.6-17.3]
**proT1**	**7.5** [7.1-7.9]

Table S2: The increase in T-cell potential after culture with DL-4 correlates with a T-lymphoid precursor phenotype

[a] Sorted CD34^{+}/CD7^{-}, ETP (CD34^{+}/CD7^{+}) and proT1 (CD34^{-}/CD7^{++}) cells from a 7-day DL-4 culture.

[b] T cell precursor: a cell able to generate CD4^{+}/CD8^{+} or CD3^{+}/TCRγδ^{+} T-cells in an OP9/DL-1 co-culture. The precursor frequency is reported as the median [interquartile range] value from three independent experiments.
